# Survival and outcomes for stroke survivors living in care homes: a prospective cohort study

**DOI:** 10.1093/ageing/afab140

**Published:** 2021-07-06

**Authors:** Amanda Clery, Finbarr C Martin, Patrick Redmond, Iain Marshall, Christopher McKevitt, Catherine Sackley, Jill Manthorpe, Charles Wolfe, Yanzhong Wang

**Affiliations:** Department of Population Health Sciences, School of Population Health and Environmental Sciences, King’s College London, London, UK; Department of Population Health Sciences, School of Population Health and Environmental Sciences, King’s College London, London, UK; Department of Population Health Sciences, School of Population Health and Environmental Sciences, King’s College London, London, UK; Department of Population Health Sciences, School of Population Health and Environmental Sciences, King’s College London, London, UK; Department of Population Health Sciences, School of Population Health and Environmental Sciences, King’s College London, London, UK; Department of Population Health Sciences, School of Population Health and Environmental Sciences, King’s College London, London, UK; National Institute for Health Research Policy Research Unit in Health and Social Care Workforce, King’s College London, London, UK; Department of Population Health Sciences, School of Population Health and Environmental Sciences, King’s College London, London, UK; National Institute for Health Research Biomedical Research Centre, Guy’s and St Thomas’ NHS Foundation Trust, London, UK; National Institute for Health Research Collaboration for Leadership in Applied Health Research and Care South London, London, UK; Department of Population Health Sciences, School of Population Health and Environmental Sciences, King’s College London, London, UK; National Institute for Health Research Biomedical Research Centre, Guy’s and St Thomas’ NHS Foundation Trust, London, UK; National Institute for Health Research Collaboration for Leadership in Applied Health Research and Care South London, London, UK

**Keywords:** care home, stroke survivor, survival, stroke outcome, older people

## Abstract

**Background:**

Stroke survivors living in care homes require high levels of support with everyday living. The aims of this study were to describe the survival, health status and care received by stroke survivors living in care homes at 1-year post-stroke, compared with those in their own homes.

**Methods:**

A total of 3,548 stroke survivors with a first ever stroke between 1998 and 2017 in the South London Stroke Register were identified for survival analysis. A total of 2,272 were included in the 1-year follow-up analysis. Cox regression and Kaplan–Meier plots were used to describe survival, stratified into four 5-year cohorts. Health status, medications and rehabilitation received at 1-year post-stroke were compared using descriptive statistics.

**Results:**

Over the 20-year period, survival improved for stroke survivors discharged to their own home (*P* < 0.001) but not for those discharged to care homes (*P* = 0.75). Care home residents were highly disabled (median Barthel index: 6/20, interquartile range: 2–10). Rates of secondary stroke prevention medications at 1-year follow-up increased over time for care home residents, including antiplatelets from 12.3 to 38.1%, although still lower than for those in their own homes (56.3%). Speech and language problems were common in the care home population (40.0%), but only 16% had received speech and language therapy.

**Conclusions:**

Rates of secondary stroke prevention prescribing increased over 20 years but remained lower in care home residents. The lower levels of rehabilitation received by stroke survivors in care homes, despite their higher levels of disability, suggest a gap in care and urgent need for restorative and/or preventative rehabilitation.

## Key Points

- Survival improved for stroke survivors discharged to their own home but not for those discharged to care homes.
- Rates of secondary stroke prevention prescribing increased over 20 years but remained lower in care home residents.
- High mortality for stroke survivors in care homes, along with differences in the care compared with those at own home.

## Introduction

Many stroke survivors have life-long disability. Rehabilitation and long-term interventions enable stroke survivors to regain functional abilities, mitigate the impact of lost function and reduce the likelihood of recurrent strokes.

Recent estimates suggest that 8–10% of stroke survivors leaving hospitals in the United Kingdom are discharged to care homes [1]. Research has reported a reduction over 20 years in the proportion of hospitalised stroke survivors discharged to care homes. Higher stroke severity and disability are more common among cohorts discharged to care homes [2], suggesting an increased need for support. In general, the needs of care home residents differ from those at home, and there is evidence of inequalities in healthcare, including poorer access compared with their counterparts not living in care homes [3, 4].

Stroke survivors living in care homes or elsewhere often have specific needs requiring specialised care [5]. The past 20 years have seen huge improvements in stroke care and support for stroke survivors in developed countries, including acute stroke care, secondary prevention and rehabilitation, leading to improved long-term outcomes [6, 7]. England’s National Institute for Health and Care Excellence guidelines for stroke rehabilitation specifically highlight the requirement for stroke survivors living in care homes to receive the same standard of treatment and appropriate equipment as those in their own homes [8]. Yet, a 2015 survey identified that as many as 60% of participating care homes could not follow these guidelines and many care home staff lacked stroke awareness [9, 10].

The aim of this study was to assess the survival patterns, health status and care received by stroke survivors living in care homes 1-year post-stroke, and the association of these to age or disability 7 days post-stroke, in order to better understand the needs in this vulnerable group.

## Methods

### Study setting

The South London Stroke Register (SLSR) is an ongoing, prospective, population-based register that has recorded all first ever stroke patients since 1 January 1995 in a defined geographical area within Lambeth and Southwark, inner-city South London, United Kingdom. Case ascertainment has been estimated at 80% [11]. Cases are ascertained from multiple sources including hospital records and general practices (GPs). Follow-up data are collected for patients at 3 months and annually post-stroke. For those living in care homes, this is typically collected via a family or staff member who knows the resident well. For those less severely disabled, assessments are conducted with the resident themselves. Details of data collection are described elsewhere [12].

Lambeth DataNet (LDN) is an electronic health records database containing data on diagnoses, consultations and prescriptions from 45 GP practices in Lambeth [13]. Approximately 50% of SLSR participants are registered with a GP in Lambeth and can therefore be linked to LDN for additional healthcare data.

### Participants

#### Inclusion criteria

Patients registered in the SLSR who had their first ever stroke (defined according to World Health Organization criteria) between 1 January 1998 and 31 December 2017 were included. Anyone discharged from hospital to a care home or who was living in a care home at their 3-month post-stroke follow-up was defined as a care home resident. Care homes provide accommodation and long-term care, some with nurses on site. Most residents live there permanently, a small proportion being short-term for respite care.

#### Exclusion criteria

For the survival analysis, patients were excluded if they were not living in their own home or a care home when discharged from hospital or at 3-month follow-up for community cases. For the clinical descriptive analyses of health status and care received at 1-year follow-up, patients were excluded if they had died, were lost to follow-up or were no longer living in a care home or their own home.

### Variables

For the survival analysis: year of stroke, discharge location, age at time of stroke, sex, ethnicity, stroke subtype and modified Barthel index at 7-day post-stroke.

For the clinical description analysis:

Demographics: year of stroke, age at time of stroke, sex, ethnicity (White, Black and other, using 2001 UK Census categories).

At 1-year follow-up:

- Functional abilities: modified Barthel index (0–20) [14], Frenchay activities index, a measure of instrumental activities (0–45) [15], Medical Outcomes Study 12-item short form (SF-12) physical and mental domains;
- speech and language deficits (collected from 2008 onwards);
- cognitive impairment (Mini-Mental State Examination score <24 before 2000, or Abbreviated Mental Test score <8 from 2000 onwards);
- co-morbidities: depression (diagnosed; collected from 2008 onwards), anxiety and depression (measured using Hospital Anxiety and Depression Scale score), hypertension, diabetes, atrial fibrillation (AF), high cholesterol;
- secondary stroke prevention medications: total number of medications prescribed (of the following), anticoagulation (if diagnosed with AF), any antiplatelets including aspirin, antihypertensives (if diagnosed with hypertension), antidiabetic medication (if diagnosed with diabetes), statins (if diagnosed with high cholesterol);
- mental health-related medications: any antidepressants, any anti-dementia medication;
- rehabilitation received: speech and language therapy, physiotherapy or occupational therapy received since the SLSR 3-month follow-up visit (collected from 2008 onwards only).

LDN: palliative care, identified using Read codes (Supplementary Table S1).

### Statistical analyses

#### Survival analysis

Survival time was defined as the date of discharge to the date of death, or censored on 15 January 2019. For patients with no recorded date of discharge, this was imputed based on the average time from date of stroke to date of discharge from their location at discharge and the year group of stroke. Kaplan–Meier curves were constructed to estimate the survival function stratified by discharge location, year of stroke (four 5-year cohorts), age at time of stroke (≤65 years old or >65 years) and Barthel index (<15, dependent, and ≥15, independent). Log-rank tests were used to assess differences in survival.

Cox regression models were built to assess mortality in stroke survivors discharged to care homes or their own home, both crude and adjusted for year of stroke, discharge location, age, sex, ethnicity, stroke subtype and post-stroke disability (Barthel index). All patients with missing data in any of these variables were excluded, and a complete case analysis was conducted. Hazard ratios, their 95% confidence intervals and *P*-values were estimated.

#### Clinical description analysis

Descriptive statistics were used to summarise demographics, functional abilities, speech and language and cognitive impairments, co-morbidities, rates of secondary stroke prevention prescribing and rehabilitation received, stratified by year and living location at follow-up. Where SLSR patients had linkage to LDN, we described palliative care provision in those living in care homes or their own homes.

#### Missing data and losses to follow-up

**Figure 1:**
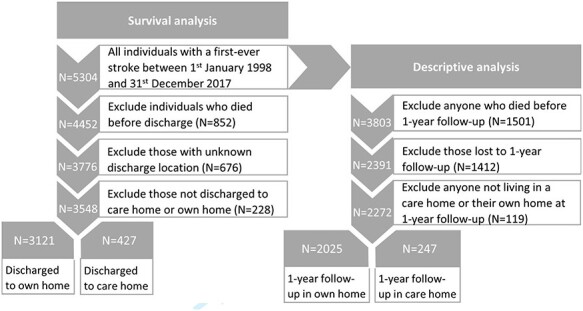
Flowchart of participants through the study.

The count and percentage of missing data was summarised for all variables. Losses to follow-up were described in the flow chart of the study (Figure 1), and a comparison was made between the characteristics of those lost and not lost to follow-up.

## Results

### Survival analysis

A total of 5,304 patients with a first ever stroke between 1 January 1998 and 31 December 2017 were identified. Of these, 852 (16.1%) died before discharge from hospital and 676 had unknown discharge location and were excluded. Of those with a known discharge location, 427 (11.3%) were discharged to care homes and 3,121 (82.7%) to their own homes (Figure 1).

**Figure 2:**
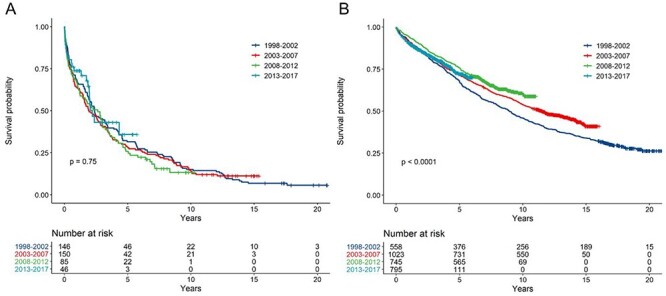
Kaplan–Meier curve for survival, stratified by year of stroke for: (A) those discharged to care homes post-stroke, *N* = 427 and (B) those discharged to their own home post-stroke, *N* = 3,121.

At time of censoring, 364 (85.2%) of the care home group had died, their median survival time being 1.8 years. In the group discharged to their own home, 1,377 (44.1%) had died, median survival being 3.9 years. Between 1998 and 2017, survival improved for those discharged to their own homes but not for the care home group (*P* < 0.0001 and *P* = 0.75, respectively, Figure 2).

**Figure 3:**
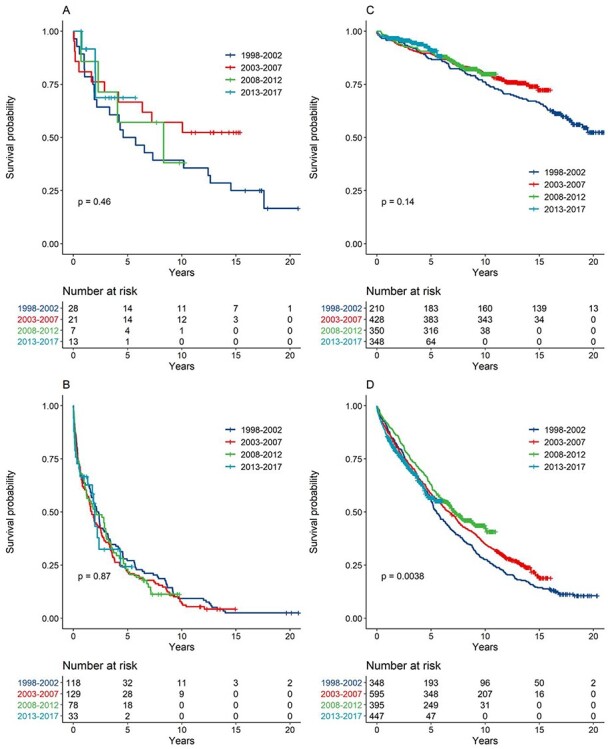
Kaplan–Meier curves for survival, stratified by year of stroke, discharge location and age: (A) discharged to care homes and 65 years old or younger, *N* = 69; (B) discharged to care homes and over 65 years old, *N* = 358; (C) discharged to their own home and 65 years old or younger, *N* = 1,336; and (D) discharged to their own home and over 65 years old, *N* = 1,785.

For those 65 and younger, there were no improvements in survival whereas; for those 65 plus, there were significant survival improvements for the own home group (*P* = 0.0038) but not the care home group (*P* = 0.87, Figure 3).

Improvements were seen in both population groups for the more independent (defined by BI (Barthel index) ≥15; Supplementary Figure S1), although sample size in the care home group was small. No improvements in survival were see09t fuyn for those more disabled (BI <15).

Cox regression analyses to assess the characteristics associated with mortality were limited to 3,078 of the 3,548 (87%) stroke survivors, due to missing data. These 3,078 comprised 363 (85%) of total care home cohort and 2,715 (87%) of those discharged home. Those discharged home were less likely to die than those discharged to care homes after adjustment for year of stroke, age, sex, ethnicity, stroke subtype and 1-week post-stroke Barthel index (adjusted HR (Hazard ratio): 0.69, 95% CI: 0.60–0.81; Table 1).

**Table 1:** Characteristics associated with mortality in stroke survivors discharged to care homes or own home, crude and adjusted for all other characteristics in the table (*N* = 3,078, events = 1,481)

	Number of events (%)	Crude hazard ratio (95% CI)	Adjusted hazard ratio (95% CI)	*P*-value
Year of stroke (versus 1998–2002)				
2003–07	611 (59.2)	0.77 (0.68–0.87)	0.83 (0.73–0.94)	0.005
2008–12	293 (40.0)	0.63 (0.54–0.73)	0.75 (0.64–0.88)	<0.001
2013–17	164 (21.2)	0.63 (0.52–0.76)	0.85 (0.70–1.03)	0.091
Discharge to own home (versus care home)	1,172 (43.2)	0.29 (0.26–0.33)	0.69 (0.60–0.81)	<0.001
Age at time of stroke	1,481 (100.0)	1.07 (1.07–1.08)	1.07 (1.06–1.07)	<0.001
Female (versus male)	742 (51.7)	1.26 (1.14–1.39)	0.85 (0.77–0.95)	0.004
Ethnicity (versus White)				
Black	318 (33.4)	0.52 (0.46–0.59)	0.79 (0.69–0.89)	<0.001
Other	80 (36.4)	0.51 (0.41–0.64)	0.64 (0.51–0.80)	<0.001
Haemorrhagic (versus ischaemic)	157 (34.9)	0.58 (0.49–0.68)	0.80 (0.68–0.95)	0.011
Barthel index 7-day post stroke	1,481 (100.0)	0.94 (0.93–0.94)	0.96 (0.95–0.97)	<0.001

### Clinical description analyses

#### Demographics

Of 5,304 stroke patients, 3,803 were alive at their 1-year follow-up, 1,412 (37%) were lost to follow-up and 119 were neither in their own home or a care home, leaving 2,272 included in the analysis, 247 in care homes and 2,025 in their own homes (Figure 1).

In the 2014–18 cohort, the mean age for care home resident stroke survivors was 75.2 compared with 67.0 years for those in their own homes. More care home residents were female, 52 versus 46%. Proportions of different ethnicities changed over the period, consistent with the changing local population profile, with a gradual increase of Black, older people, and decrease of White, older people. Distributions between care home and at home were similar across ethnicities, summarised in Table 2.

**Table 2:** Prevalence of the characteristics of stroke survivors at 1-year follow-up, stratified by year of follow-up and living location

	Care home				Own home			
	1999–2003 (*N* = 75)	2004–08 (*N* = 97)	2009–13 (*N* = 50)	2014–18 (*N* = 25)	1999–2003 (*N* = 499)	2004–08 (*N* = 654)	2009–13 (*N* = 460)	2014–18 (*N* = 412)
Demographics								
Age at time of stroke, years: mean (SD)	74.5 (12.6)	78.8 (10.5)	79.0 (9.8)	75.2 (13.9)	66.2 (13.4)	66.6 (14.1)	66.4 (14.9)	67.0 (14.7)
Female	45 (60.0)	52 (53.6)	32 (64.0)	13 (52.0)	219 (43.9)	274 (41.9)	218 (47.4)	189 (45.9)
Ethnicity								
White	59 (79.7)	81 (84.4)	36 (72.0)	12 (52.2)	329 (66.6)	411 (63.7)	263 (57.7)	224 (54.8)
Black	13 (17.6)	11 (11.5)	11 (22.0)	10 (43.5)	129 (26.1)	179 (27.8)	161 (35.3)	160 (39.1)
Other	2 (2.7)	4 (4.2)	3 (6.0)	1 (4.3)	36 (7.3)	55 (8.5)	32 (7.0)	25 (6.1)
Functional abilities								
Barthel index: median (IQR)	6.0 (2.0–11.0)	8.0 (2.0–14.0)	5.0 (2.0–11.0)	6.0 (2.0–10.0)	19.0 (16.0–20.0)	19.0 (17.0–20.0)	19.0 (16.0–20.0)	20.0 (17.0–20.0)
Feeding difficulties	45 (60.8)	59 (60.8)	35 (71.4)	14 (56.0)	93 (18.7)	107 (16.5)	49 (10.7)	42 (10.2)
Bladder incontinence	53 (70.7)	68 (70.1)	41 (83.7)	19 (76.0)	110 (22.2)	141 (21.8)	98 (21.8)	68 (16.5)
Bowel incontinence	42 (56.0)	57 (58.8)	36 (73.5)	19 (76.0)	57 (11.5)	75 (11.6)	65 (14.4)	40 (9.7)
Frenchay activities index: median (IQR)	2.0 (0.0–5.0)	2.0 (0.0–6.0)	0.0 (0.0–3.8)	0.0 (0.0–3.0)	18.0 (9.0–29.0)	20.0 (9.0–30.0)	20.0 (11.0–29.0)	20.0 (6.0–30.0)
Taken part in a hobby	22 (31.4)	34 (35.8)	11 (23.9)	3 (12.0)	201 (41.2)	380 (58.7)	235 (52.2)	218 (53.0)
Been to a social occasion	20 (28.2)	36 (37.9)	14 (29.2)	5 (20.0)	333 (67.7)	443 (68.5)	302 (68.2)	298 (72.3)
Been on a travel outing	26 (36.6)	23 (24.2)	15 (31.2)	4 (16.0)	271 (55.2)	346 (53.4)	313 (69.7)	234 (56.8)
Read a book	23 (32.4)	16 (16.8)	8 (16.7)	3 (12.0)	238 (48.5)	339 (52.4)	214 (47.8)	137 (33.4)
Walked outside for >15 min	12 (16.9)	12 (12.6)	6 (13.0)	1 (4.0)	377 (76.3)	456 (70.3)	351 (79.1)	299 (72.6)
Speech and language deficits	–	–	20 (40.0)	10 (40.0)	–	–	93 (20.8)	105 (25.7)
Cognitive impairment	37 (69.8)	74 (82.2)	32 (82.1)	17 (85.0)	165 (36.3)	235 (36.8)	115 (27.1)	119 (31.4)
Co-morbidities								
Depression	–	–	7 (15.9)	9 (36.0)	–	–	66 (15.7)	60 (14.7)
Hypertension	51 (71.8)	75 (77.3)	41 (82.0)	21 (84.0)	329 (68.5)	546 (83.5)	357 (77.6)	357 (86.7)
Diabetes	17 (23.9)	24 (24.7)	11 (22.0)	15 (60.0)	103 (21.3)	165 (25.2)	138 (30.0)	140 (34.0)
Atrial fibrillation	13 (18.3)	32 (33.3)	16 (32.0)	9 (36.0)	67 (13.9)	124 (19.0)	105 (22.8)	137 (33.3)
High cholesterol	24 (33.3)	47 (48.5)	28 (56.0)	20 (80.0)	209 (44.0)	427 (65.3)	284 (61.7)	331 (80.3)
Secondary stoke prevention medications								
Total no: median (IQR)	1.0 (1.0–2.8)	3.0 (2.0–4.0)	3.0 (2.0–3.0)	3.0 (1.0–3.0)	2.0 (1.0–3.0)	3.0 (2.0–4.0)	3.0 (2.0–3.0)	3.0 (2.0–3.0)
Anticoagulation (if AF)	1 (7.7)	8 (25.0)	3 (21.4)	4 (50.0)	28 (41.8)	51 (41.1)	53 (51.0)	72 (54.1)
Antiplatelets	9 (12.3)	25 (25.8)	23 (47.9)	8 (38.1)	65 (13.1)	197 (30.4)	239 (52.8)	220 (56.3)
Aspirin	45 (60.8)	76 (78.4)	19 (39.6)	1 (4.8)	315 (63.1)	409 (63.0)	182 (40.2)	39 (10.1)
Antihypertensives (if hypertensive)	28 (57.1)	45 (60.0)	12 (30.8)	9 (52.9)	264 (83.5)	442 (81.5)	195 (55.4)	182 (53.8)
Antidiabetic (if diabetic)	12 (70.6)	20 (83.3)	4 (36.4)	5 (45.5)	60 (58.3)	127 (77.4)	88 (64.7)	97 (74.0)
Statins (if high cholesterol)	10 (41.7)	30 (63.8)	23 (82.1)	13 (81.2)	126 (60.3)	357 (84.2)	235 (83.9)	273 (87.5)
Mental health-related medications								
Antidepressants	0 (0.0)	27 (27.8)	19 (38.0)	14 (56.0)	0 (0.0)	69 (10.6)	66 (14.4)	69 (16.8)
Anti-dementia	0 (0.0)	0 (0.0)	3 (6.0)	2 (8.0)	0 (0.0)	0 (0.0)	2 (0.4)	6 (1.5)
Rehabilitation received								
Speech and language therapy	–	–	9 (20.0)	4 (16.0)	–	–	65 (14.4)	32 (7.9)
Physiotherapy	–	–	13 (28.3)	5 (20.0)	–	–	163 (35.7)	115 (28.0)
Occupational therapy	–	–	8 (17.8)	1 (4.2)	–	–	83 (18.5)	37 (9.1)

Data are presented as numbers (*n*) and percentages (%), unless otherwise specified. Where missing, data were not collected for these time periods.

#### Health status

Consistently across 20 years, those in care homes were more disabled than those in their own homes at 1-year post-stroke (e.g. median BI: 6/20 (interquartile range (IQR) 2–10) and 20/20 (IQR 17–20), respectively, in 2014–18). Difference included rates of bowel incontinence: 76.0% of care home residents in the 2014–18 cohort (an increase from 56.0% in 1999–2003) compared with 9.7% of those living in their own homes (a decrease from 11.5% in 1999–2003). The Frenchay activities indexes were also higher (more able) for those living in their own homes, some individual items scores tending to increase over time for those living at home and decrease for care home residents. For example, the proportion of stroke survivors at home and active in a hobby in the 3 months pre-assessment increased from 41.2% in 1999–2003 to 53.0% in 2014–18, but dropped from 31.4 to 12.0% for care home residents.

#### Care received

Rates of prescriptions of secondary stroke preventative medicines increased over the 20-year period in both groups, particularly for anticoagulants, antiplatelet agents and statins, but were higher for those in their own homes for all medications in all time periods except for aspirin during 2014–18. Antidepressant and anti-dementia prescriptions were higher in care home residents. In 2014–18, 56.0% of care home residents were prescribed antidepressants.

Finally, over the 10 years between 2009 and 2018, there was a trend towards less rehabilitation activity for stroke survivors living in both care homes and own homes during the 9 months to the 1-year follow-up. However, these results are based on a small sample size in each subgroup, particularly in care homes, summarised in Table 2.

LDN linkage was completed for 134 (54%) stroke survivors living in care homes and 1,046 (52%) at home. Seven (5.2%) of those in care homes had advance care or palliative care plans in place, compared with 12 (1.2%) of those at home; these numbers being too small to draw any firm conclusions. However, those with palliative care plans tended to be older and more disabled.

### Missing data

Missing data for the variables in the 2,272 stroke survivors identified for analysis are summarised in the Supplementary Table S3. Most variables had missingness levels <5%, except for cognitive impairment, which was 20% in those in care homes and 8% for those living at home. Some of those living in care homes had very high levels of missingness (>50%) compared with only around 10% in those living in their own homes. Due to this skewed missingness, we did not analyse data on the following outcomes: SF-12 physical and mental scores, and anxiety and depression measured by the Hospital Anxiety and Depression Scale score.

## Discussion

### Clinical trajectories—survival and health status at 1 year

Over 20 years, stroke survivors living in their own homes 1-year post-stroke were on average younger, more likely male, and survived longer than those entering and remaining in care homes, median survival time being about double. The difference remained after adjustment for demographic variables and functional ability at 7-day post-stroke. Survival rates improved over time only for those at home, significantly in those aged 65 plus. For both groups, improvement was evident only in those less disabled 1-week post-stroke. This probably accounts for the overall lack of survival improvement for those in care homes, as 93% were more disabled (BI <15).

One-year post-stroke, care home survivors were on average less able in personal and instrumental care activities and had higher rates of impairments associated with stroke, such as bowel incontinence, speech, language and cognitive impairments. These differences persisted over time. In contrast to care home residents, functional abilities of those at home showed general improvements over time, albeit small. Rates of depression were consistently higher in care homes residents.

These characteristics of stroke survivors living in care homes 1-year post-stroke remain similar to the characteristics of those discharged to care homes, compared with their counterparts living in their own homes [2, 16].

### Care received—medications and rehabilitation

Although prescription rates for stroke secondary prevention medications increased for both groups over the 20 years, they were persistently higher for those at home. We did not investigate clinical decision making, and therefore we cannot infer whether or not this is appropriate care in this population. Differences in potential benefits, risks and burdens of medications may lead to less initiation or more frequent deprescribing among care home residents, for example, for antihypertensives [17]. Nevertheless, rates of some prescriptions were lower than is likely to be optimal, e.g. only half of care home residents with AF were on anticoagulant. This confirms other reports [18] and merits future investigation. Medications for depression and dementia were higher for care home residents, consistent with likely higher rates of these conditions in care home residents [19].

Rates of rehabilitation input were generally low (e.g. the highest rates were between 20 and 35% for physiotherapy) and reduced for all three types assessed.

### Limitations

Internal generalisability is impacted by the overall participant loss to follow-up rate of 37%. Those lost to 1-year follow-up were younger, more likely to have been discharged home and more functionally able (Supplementary Table S4). Since these characteristics are associated with better health outcomes, the differences found between those living in their own homes and those in care homes may be underestimated. Conclusions from the analyses of time trends among those discharged to care homes are limited by small numbers. Discharge destination was also unknown for 676 (15%) stroke survivors, some of which were re-identified by our SLSR fieldworkers in the 1-year follow-up analysis. However, this highlights the need for clear follow-up pathways of stroke patients for both care and research purposes.

High levels of likely non-random missing data relating to cognition and mental health precluded meaningful analyses. These variables require answers from residents themselves, not proxy or assisted reporting. Thus, our data are biased towards residents with better cognitive or communication ability. This should spur more research in developing alternative ways to assess at scale mental health domains in care home residents with limited capacity.

### Implications

Rates of discharge of this population of stroke survivors to a care home were only slightly higher than the national average, so the case-mix is likely fairly representative [1, 2]. Health status trajectories in care homes generally, and for stroke survivors specifically, are inadequately documented to enable comparison with these cohorts. Future research to enable such analyses could be made possible with data linkage. We show successful linkage to the LDN for the SLSR population—this presents opportunities for greater characterisation of morbidity (e.g. electronic frailty index) [20]. It also addresses the need for a national minimum data set in care homes, which would allow for more comprehensive research in this often-overlooked population [21].

The improving functional trajectories for stroke survivors at home but not in care homes may reflect widening baseline differences in factors not captured in the parameters studied. Differential trajectories of disability and participation in activities may reflect that care home life does not maximise retention of functional ability or social participation.

There are several possible explanations for the likely gaps in care for stroke survivors living in care homes. The divergence of rehabilitation input and social activities further diverged between general community and care home residents during this period. These findings may not be typical as there is geographical variation in access to healthcare, but observed rates of rehabilitation input observed were surprisingly low. Enabling and encourage social participation to reduce isolation is likely to improve well-being. Depressive symptoms are associated with restricted participation [22]. Given the high level of depression diagnosed in this population, an understanding of what might help prevent or reduce this is warranted. A successful approach may need to address system barriers and facilitators as well as individual factors.

The best ways of providing stroke-orientated rehabilitation for the care home population are not yet clear. Many rehabilitation studies have included less impaired participants: care home residents are under-represented [23–25]. Care home-specific studies of interventions known to be effective in community settings show small and inconsistent effectiveness [26]. Stroke-specific occupational therapy-led rehabilitation interventions aimed at maintaining functional activity and reducing further health risks from inactivity of care home resident stroke survivors were ineffective [27]. This uncertainty may undermine service commitment to rehabilitation with stroke survivors in care homes.

## Conclusion

Stroke survivors living in care homes have complex needs, which requires complex clinical decision making. This study reveals high mortality for stroke survivors in care homes, along with differences in the care they receive compared with stroke survivors living in their own homes. This vulnerable group needs individualised care; therefore, the reasons behind these gaps in care calls for more detailed understanding of care home decision making. This could be addressed with improved data collection and linkage in care homes.

## Supplementary Material

aa-21-0105-File002_afab140
