# Supplementary material for: Survival and outcomes for stroke survivors living in care homes: a prospective cohort study
Source: Age Ageing. 2021 Jul 6;50(6):2079–87. doi: 10.1093/ageing/afab140 (PMC8581388; doi:10.1093/ageing/afab140)

SUPPLEMENTAL MATERIAL

Survival and outcomes for stroke survivors living in care homes: a prospective cohort study

List of Contents

Table I: Read codes for palliative care

Table II. Prevalence of the characteristics of stroke survivors at 1-year follow-up, stratified by living location and palliative care provision

Table III. Missing data in all outcome variables at 1-year follow-up, stratified by year of follow-up and living location

Table IV. Baseline characteristics of those lost to follow-up compared to those not lost to follow-up

Figure I. Kaplan-Meier curves for survival, stratified by year of stroke, discharge location, and Barthel index (BI) at 7-days post-stroke

Table I: Read codes for palliative care. A combination of text mining and guideline documents e.g. NHS Derbyshire County and NHS Derby City. Derbyshire End of Life Care Guidance: A pathway for supporting people in the last year of life.

8BJ1. Palliative Treatment
8BAP. Specialist Palliative care - QOF
8CM3. Palliative care plan review
8H7g. Referral to palliative care service - QOF
8CM1. On GSF - QOF
9Nh0. Under the care of community palliative care team
8BAR. Specialist palliative care treatment - inpatient
8BAT. Specialist palliative care treatment – outpatient
8HH7. Referred to community specialist palliative care team
8CN1 - preferred place of death discussed with patient
8CN0 - preferred place of death discussed with significant other
ZV57C - palliative care - QOF
9ke - palliative enhanced services administration
9e2 - GP OOH handover form completed
9e00 - GP OOH service notified of cancer care plan
1R1 - not for resuscitation Xa9tT not for resuscitation
8o7 carer support Xa1oA carer support
8BMM - issue of palliative care anticipatory medication box
8GA0 – crisis intervention
1Z01.Terminal illness – late stage
8BAS. Specialist palliative care treatment – daycare
8BAe. Anticipatory palliative care
8H6A. Refer to terminal care consult
8H7L. Refer for terminal care
9EB5. DS 1500 Disability living allowance (terminal care) completed
**Gold Standards Framework (GSF) Register**
8CM1. 8CM10 8CM11 8CM12 8CM13 8CM15 8CM16 8CM17 8CM18
**Sharing end of life care/LPA**
9Ng7. 9Nu6. 9Nu8. 9Nu9. 9Nu90
**LPA/Preferred place of death/resuscitation**
8CM1. 8CM10 8CM11 8CM12 8CM13 8CM15 8CM16 8CM17 8CM18 8CME 9NgH 9NgE 9NgJ 9NgG 9NgG0 9W4 9W5 9W8 9W80 8Ce8 8Ce9 8CeA 8CeB 8CN1 94ZB 94Z6 94Z7 94Z8 94ZD 94Z1. 94Z2. 94Z3. 94Z4. 94Z5. 94Z9. 94ZA. 94ZE. 94ZF. 8Ce0. 8Ce1. 8Ce2. 8Ce3. 8Ce4. 8Ce5. 8Ce6. 8Ce7. 1R00. 1R10. 67P0 8CN7
**Notification to Out of Hours re: palliative care**
9G8 9e02
**Anticipatory meds**
8B2a
**"End of life":**
8CMg. 8BA2. 8CMW3 8CMj.
**“Palliative”**
9NlJ. 8HH7. 8CM1. 8H7g. 9NgzS 8BAP. 9NgD. 8HgX. 9Nh0. 8BAe. 8Hg0. 8BAT. 8B2a. EMISNQPR3 8BAN. 8IEE. 9hB0. 8BMM. 8BMM. EMISNQON15 9367. 9NNd. 8BAR. 9NNf0 9c0P. 9c0L0 9e02. 8BAS. EMISNQPA133 EMISNQCO135 9hB.. 9hB1. EMISNQHE22 EMISNQIN259

Table II. Prevalence of the characteristics of stroke survivors at 1-year follow-up, stratified by living location, and whether they were identified as having palliative care provision

	**Care home**		**Own home**	
	**No palliative care (N=127)**	**Palliative care (N=7)**	**No palliative care (N=1031)**	**Palliative care (N=12)**
**Demographics**				
Mean age, years: mean (SD)	76.5 (11.8)	86.2 (5.9)	66.6 (14.3)	71.6 (12.3)
Female	74 (58.3)	3 (42.9)	476 (46.2)	6 (50.0)
Ethnicity				
White	91 (73.4)	3 (42.9)	549 (53.8)	6 (50.0)
Black	29 (23.4)	4 (57.1)	395 (38.7)	6 (50.0)
Other	4 (3.2)	0 (0.0)	77 (7.5)	0 (0.0)
**Functional abilities**				
Barthel index: median (IQR)	6.5 (2.0-13.0)	1.0 (1.0-1.5)	20.0 (17.0-20.0)	14.0 (6.8-19.2)
Feeding difficulties	77 (61.1)	7 (100.0)	139 (13.5)	4 (33.3)
Bladder incontinence	89 (70.6)	7 (100.0)	195 (19.0)	4 (33.3)
Bowel incontinence	78 (61.9)	7 (100.0)	108 (10.5)	3 (25.0)
Frenchay activities index: median (IQR)	2.5 (0.0-5.0)	0.0 (0.0-0.0)	20.0 (9.0-30.0)	0.0 (0.0-10.5)
Taken part in a hobby	41 (33.1)	0 (0.0)	530 (52.1)	4 (33.3)
Been to a social occasion	48 (38.4)	1 (16.7)	710 (69.5)	4 (33.3)
Been on a travel outing	44 (35.2)	0 (0.0)	582 (57.1)	4 (33.3)
Read a book	24 (19.2)	0 (0.0)	487 (48.0)	1 (8.3)
Walked outside for >15 mins	21 (16.9)	0 (0.0)	766 (75.2)	5 (41.7)
**Speech and language deficits**	18 (42.9)	1 (16.7)	101 (21.3)	5 (45.5)
**Cognitive impairment**	79 (74.5)	6 (85.7)	346 (36.1)	6 (50.0)
**Co-morbidities**				
Depression	9 (11.7)	4 (57.1)	89 (12.9)	1 (8.3)
Hypertension	94 (74.6)	7 (100.0)	814 (79.6)	11 (91.7)
Diabetes	37 (29.4)	4 (57.1)	285 (27.8)	5 (41.7)
Atrial fibrillation	37 (29.6)	4 (57.1)	216 (21.1)	5 (41.7)
High cholesterol	65 (51.6)	5 (71.4)	634 (62.2)	7 (58.3)
**Secondary stroke prevention medications**				
Total: median (IQR)	2.0 (1.0-3.0)	2.0 (1.5-4.0)	3.0 (2.0-3.0)	2.5 (1.0-3.0)
Anticoagulation (if AF)	10 (27.8)	1 (25.0)	109 (50.7)	2 (40.0)
Antiplatelets	30 (24.6)	3 (42.9)	363 (35.7)	6 (54.5)
Aspirin	77 (62.6)	1 (14.3)	457 (45.0)	2 (18.2)
Antihypertensives (if hypertensive)	47 (52.2)	3 (42.9)	560 (70.4)	4 (40.0)
Anti-diabetic (if diabetic)	22 (64.7)	3 (75.0)	194 (69.5)	4 (80.0)
Statins (if high cholesterol)	47 (75.8)	3 (60.0)	495 (79.6)	5 (83.3)
Mental health related medications				
Antidepressants	27 (21.6)	4 (57.1)	97 (9.5)	4 (33.3)
Anti-dementia	1 (0.8)	1 (14.3)	3 (0.3)	0 (0.0)
**Rehabilitation received**				

Speech and language therapy	8 (22.2)	0 (0.0)	61 (12.9)	2 (18.2)
Physiotherapy	12 (33.3)	0 (0.0)	155 (32.3)	4 (36.4)
Occupational therapy	8 (22.9)	0 (0.0)	79 (16.6)	0 (0.0)

Table III. Missing data in all outcome variables at 1-year follow-up, stratified by year of follow-up and living location.

	Care home				Own home			
	1999-2003	2004-2008	2009-2013	2014-2018	1999-2003	2004-2008	2009-2013	2014-2018
**Demographics**								
Age	0 (0.0)	0 (0.0)	0 (0.0)	0 (0.0)	0 (0.0)	0 (0.0)	0 (0.0)	0 (0.0)
Sex	0 (0.0)	0 (0.0)	0 (0.0)	0 (0.0)	0 (0.0)	0 (0.0)	0 (0.0)	0 (0.0)
Ethnicity	1 (1.3)	1 (1.0)	0 (0.0)	2 (8.0)	5 (1.0)	9 (1.4)	4 (0.9)	3 (0.7)
**Functional abilities**								
Barthel index	2 (2.7)	1 (1.0)	3 (6.0)	0 (0.0)	9 (1.8)	8 (1.2)	30 (6.5)	7 (1.7)
Feeding difficulties	1 (1.3)	0 (0.0)	1 (2.0)	0 (0.0)	2 (0.4)	4 (0.6)	3 (0.7)	0 (0.0)
Bladder incontinence	0 (0.0)	0 (0.0)	1 (2.0)	0 (0.0)	3 (0.6)	6 (0.9)	10 (2.2)	0 (0.0)
Bowel incontinence	0 (0.0)	0 (0.0)	1 (2.0)	0 (0.0)	3 (0.6)	6 (0.9)	8 (1.7)	0 (0.0)
Frenchay activities index	7 (9.3)	2 (2.1)	12 (24.0)	0 (0.0)	18 (3.6)	21 (3.2)	75 (16.3)	12 (2.9)
Taken part in a hobby	5 (6.7)	2 (2.1)	4 (8.0)	0 (0.0)	11 (2.2)	7 (1.1)	10 (2.2)	1 (0.2)
Been to a social occasion	4 (5.3)	2 (2.1)	2 (4.0)	0 (0.0)	7 (1.4)	7 (1.1)	17 (3.7)	0 (0.0)
Been on a travel outing	4 (5.3)	2 (2.1)	2 (4.0)	0 (0.0)	8 (1.6)	6 (0.9)	11 (2.4)	0 (0.0)
Read a book	4 (5.3)	2 (2.1)	2 (4.0)	0 (0.0)	8 (1.6)	7 (1.1)	12 (2.6)	2 (0.5)
Walked outside for >15 mins	4 (5.3)	2 (2.1)	4 (8.0)	0 (0.0)	5 (1.0)	5 (0.8)	16 (3.5)	0 (0.0)
SF-12 physical	56 (74.7)	56 (57.7)	13 (26.0)	0 (0.0)	225 (45.1)	81 (12.4)	51 (11.1)	15 (3.6)
SF-12 mental	56 (74.7)	56 (57.7)	13 (26.0)	0 (0.0)	225 (45.1)	81 (12.4)	51 (11.1)	15 (3.6)
Anxiety (HADS)	45 (60.0)	53 (54.6)	24 (48.0)	13 (52.0)	100 (20.0)	68 (10.4)	51 (11.1)	31 (7.5)
Depression (HADS)	44 (58.7)	53 (54.6)	18 (36.0)	13 (52.0)	96 (19.2)	68 (10.4)	45 (9.8)	32 (7.8)
**Speech and language deficits**	-	-	1 (2.0)	0 (0.0)	-	-	12 (2.6)	4 (1.0)
**Cognitive impairment**	22 (29.3)	7 (7.2)	11 (22.0)	5 (20.0)	44 (8.8)	16 (2.4)	36 (7.8)	33 (8.0)
**Co-morbidities**								
Depression	-	-	6 (12.0)	0 (0.0)	-	-	39 (8.5)	5 (1.2)
Hypertension	4 (5.3)	0 (0.0)	0 (0.0)	0 (0.0)	19 (3.8)	0 (0.0)	0 (0.0)	0 (0.0)
Diabetes	4 (5.3)	0 (0.0)	0 (0.0)	0 (0.0)	16 (3.2)	0 (0.0)	0 (0.0)	0 (0.0)
Atrial fibrillation	4 (5.3)	1 (1.0)	0 (0.0)	0 (0.0)	16 (3.2)	0 (0.0)	0 (0.0)	0 (0.0)
High cholesterol	3 (4.0)	0 (0.0)	0 (0.0)	0 (0.0)	24 (4.8)	0 (0.0)	0 (0.0)	0 (0.0)
**Secondary stroke prevention medications**								
Total no. of medications	1 (1.3)	0 (0.0)	0 (0.0)	0 (0.0)	0 (0.0)	1 (0.2)	0 (0.0)	1 (0.2)
Anticoagulation (if AF)	0 (0.0)	0 (0.0)	2 (12.5)	1 (11.1)	0 (0.0)	0 (0.0)	1 (1.0)	4 (2.9)
Antiplatelets	2 (2.7)	0 (0.0)	2 (4.0)	4 (16.0)	1 (0.2)	5 (0.8)	7 (1.5)	21 (5.1)
Aspirin	1 (1.3)	0 (0.0)	2 (4.0)	4 (16.0)	0 (0.0)	5 (0.8)	7 (1.5)	24 (5.8)
Antihypertensives (if hypertensive)	2 (3.9)	0 (0.0)	2 (4.9)	4 (19.0)	13 (4.0)	4 (0.7)	5 (1.4)	19 (5.3)

Anti-diabetic (if diabetic)	0 (0.0)	0 (0.0)	0 (0.0)	4 (26.7)	0 (0.0)	1 (0.6)	2 (1.4)	9 (6.4)
Statins (if high cholesterol)	0 (0.0)	0 (0.0)	0 (0.0)	4 (20.0)	0 (0.0)	3 (0.7)	4 (1.4)	19 (5.7)
**Mental health related medications**								
Antidepressants	4 (5.3)	0 (0.0)	0 (0.0)	0 (0.0)	12 (2.4)	1 (0.2)	1 (0.2)	1 (0.2)
Anti-dementia	4 (5.3)	0 (0.0)	0 (0.0)	0 (0.0)	12 (2.4)	1 (0.2)	1 (0.2)	1 (0.2)
**Rehabilitation received**								
Speech and language therapy	-	-	5 (10.0)	0 (0.0)	-	-	10 (2.2)	6 (1.5)
Physiotherapy	-	-	4 (8.0)	0 (0.0)	-	-	3 (0.7)	2 (0.5)
Occupational therapy	-	-	5 (10.0)	1 (4.0)	-	-	11 (2.4)	5 (1.2)

Table IV. Baseline characteristics of those lost to follow-up compared to those not lost to follow-up

	**Not lost to follow-up N=2391**	**Lost to follow-up N=1412**	**P-value**
**Discharge location**			<0.001
**Care home**	211 (9.5)	84 (7.2)	
**Private home**	1887 (85.3)	1006 (85.8)	
**Other**	114 (5.2)	76 (6.5)	
**Mean age at stroke (SD)**	68.07 (14.28)	63.77 (15.88)	<0.001
**7-day post-stroke BI<15**	866 (41.8)	455 (37.8)	0.024

Figure I. Kaplan-Meier curves for survival, stratified by year of stroke, discharge location, and Barthel index (BI) at 7-days post-stroke: (A) discharged to care homes and BI≥15, N=26 (B) discharged to care homes and BI<15, N=341 (C) discharged to their own home and BI≥15, N=1707 and (D) discharged to their own home and BI<15 N=1043.

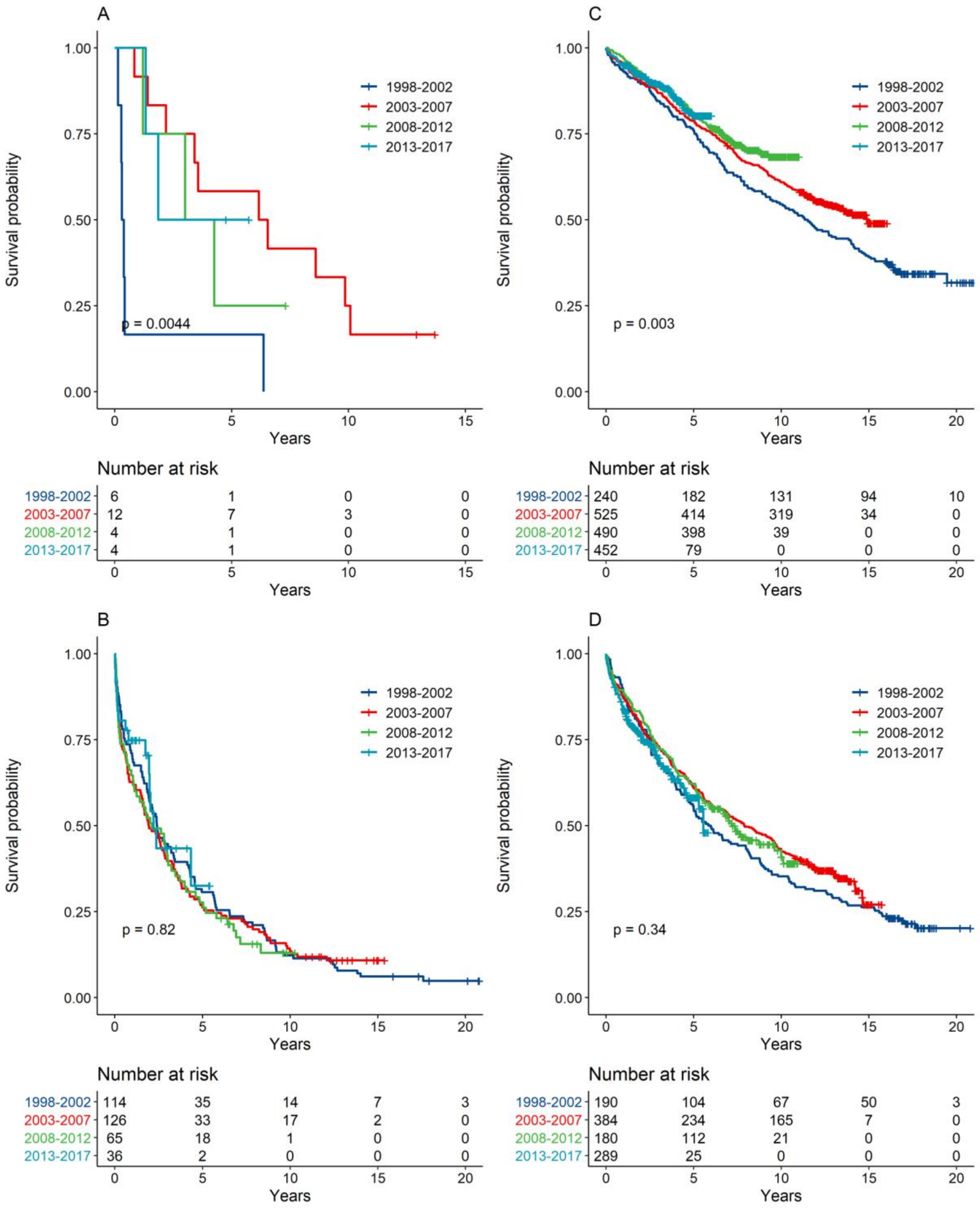

Supplement: aa-21-0105-File002_afab140 [file aa-21-0105-file002_afab140.pdf]
